# Edge/Fog Computing Technologies for IoT Infrastructure

**DOI:** 10.3390/s21093001

**Published:** 2021-04-25

**Authors:** Taehong Kim, Seong-eun Yoo, Youngsoo Kim

**Affiliations:** 1 School of Information and Communication Engineering, Chungbuk National University, Chungbuk 28644, Korea; taehongkim@cbnu.ac.kr; 2 School of Computer and Communication Engineering, Daegu University, Gyeongsan 38453, Korea; seyoo@daegu.ac.kr; 3 Military Development Research Center, Korea Institute for Defense Analyses, Seoul 02455, Korea

The prevalence of smart devices and cloud computing has led to an explosion in the amount of data generated by IoT devices. Moreover, emerging IoT applications, such as augmented and virtual reality (AR/VR), intelligent transportation systems, and smart factories require ultra-low latency for data communication and processing. Fog/edge computing is a new computing paradigm where fully distributed fog/edge nodes located nearby end devices provide computing resources. By analyzing, filtering, and processing at local fog/edge resources instead of transferring tremendous data to the centralized cloud servers, fog/edge computing can reduce the processing delay and network traffic significantly. With these advantages, fog/edge computing is expected to be one of the key enabling technologies for building the IoT infrastructure.

Aiming to explore the recent research and development on fog/edge computing technologies for building an IoT infrastructure, this Special Issue collected several dozens of submissions and finally published 10 articles (one review and nine full-length articles) after the thorough review process. The selected articles cover diverse topics such as resource management, service provisioning, task offloading and scheduling, container orchestration, and security on edge/fog computing infrastructure, which can help to grasp the recent trends, as well as the state-of-the-art algorithms of the fog/edge computing technologies.

The review article “Resource Management Techniques for Cloud/Edge and Edge Computing: An Evaluation Framework and Classification” authored by Adriana Mijuskovic et al. [1] provides a comprehensive review on the resource management techniques applied for cloud, fog, and edge computing. They first classify various techniques into six classes, such as discovery, load-balancing, off-loading, deployment, QoS (quality of service), and energy management according to the goal and methodologies, and then analyze each algorithm from the viewpoint of the resource management types, such as resource allocation, workload balance, resource provisioning, and task scheduling. 

The next article “Optimal Service Provisioning for the Scalable Fog/Edge Computing Environment” written by Jonghwa Choi and Sanghyun Ahn [2] proposes a service provisioning algorithm to optimally place service images for the service demands obtained from the prior time interval. They propose two heuristic algorithms such as MC-SP (maximal coverage service provisioning) and FC-SP (flexible coverage service provisioning) based on the logical fog network. The evaluation results show better performance than the on-demand resource provisioning mechanism in terms of the number of service image placements and the network cost per service request.

In the article [3], Md Delowar Hossain et al. propose a fuzzy decision-based task offloading management (FTOM) scheme in multi-tier MEC (multi-access edge computing) systems. The FTOM scheme selects an optimal target node to offload the task based on the server resource status and network condition, where it is designed to prefer the local or nearby servers by considering the latency sensitiveness of the tasks. The performance evaluations show that the proposed scheme outperforms the existing scheme in terms of the successful task offloading rate and the task completion time. 

Another article, “Dynamically Controlling Offloading Thresholds in Fog Systems”, authored by Faten Alenizi and Omer Rana [4], proposes an offloading scheme that can dynamically adjust the threshold to offload the tasks based on two algorithms such as the dynamic task scheduling (DTS) and dynamic energy control (DEC). The experimental results prove that the delay and the throughput can be improved by dynamically adjusting the ratio of tasks to be processed locally and the tasks to be offloaded based on the resource status of the local fog node and its neighboring nodes.

The article “Deep Reinforcement Learning-Based Task Scheduling in IoT Edge Computing” authored by Shuran Sheng et al. [5] formulates a Markov decision process (MDP) model for the resource allocation and task scheduling problem in the IoT edge computing environment, where computation-intensive tasks are scheduled and processed by an individual virtual machine with heterogeneous capacity. They apply deep reinforcement learning (DRL) to solve the MDP problem, and they demonstrate that the proposed algorithm achieves better performance in terms of task success ratio and the cumulative task satisfaction degree than the benchmark methods.

The next article “Efficient Implementation of NIST LWC ESTATE Algorithm Using OpenCL and Web Assembly for Secure Communication in Edge Computing Environment” written by Bosun Park and Seog Chung Seo [6] proposes methods to efficiently operate the ESTATE crypto algorithm using Web Assembly and OpenCL parallel processing for securing edge computing applications. The experimental evaluation shows that the proposed mechanism is five times faster than the C implementation by simultaneously encrypting data to be transmitted to multiple devices through OpenCL parallel processing. 

In the article “Identification of IoT Actors” [7], Suada Hadzovic et al. focus on clarifying the identification of IoT actors used in one thousand IoT-related standards. Based on the five layers of computing paradigm such as cloud, fog, edge, mist, and dew computing, they define the IoT model by mapping diverse IoT actors as well as IoT components such as a thing, gateway, service, user, etc., which is expected to provide a clearer clarification of the blurred definition of IoT actors and their relationship.

The article “Hyper-Angle Exploitative Searching for Enabling Multi-Objective Optimization of Fog Computing” authored by Taj-Aldeen Naser Abdali et al. [8] formulates a novel fog computing optimization framework to achieve multiple objectives, such as time latency, energy consumption, energy distribution, renting cost, and reliability at the same time. They propose an HAES (hyper-angle exploitative searching) algorithm to prioritize solutions within the same rank. The evaluation results show that HAES outperforms the benchmark protocols in terms of various performance metrics, such as the hyper volume measure, the number of non-dominated solutions, generational distance measure, delta metric, and the set of coverage measure.

The last two articles focus on the Kubernetes container orchestration platform. The article “Horizontal Pod Autoscaling in Kubernetes for Elastic Container Orchestration” authored by Thanh-Tung Nguyen et al. [9] addresses that the Kubernetes provides diverse autoscaling mechanisms to support the high availability and scalability of the services. They investigate HPA (horizontal pod autoscaling) through diverse experiments. By including the comparison of the difference between Kubernetes resource metrics (KRM) and Prometheus custom metrics (PCM), they provide a detailed analysis and lessons to optimize the performance of HPA in the Kubernetes cluster. 

The next article, “Balanced Leader Distribution Algorithm in Kubernetes Clusters”, authored by Nguyen Dinh Nguyen and Taehong Kim [10], focuses on the stateful applications requiring a strong consistency of data among the replicas. The authors address that the leader-based consistency mechanisms may lead to a workload imbalance problem that a specific node with multiple concentrated leaders suffers from the heavy load due to its inherent design. They propose a balanced leader distribution algorithm to overcome the problem, and the experimental evaluations prove that distributing the leaders throughout nodes in the cluster improves the overall throughput of the cluster as well.
